# Multi-Sensor Monitoring, Intelligent Control, and Data Processing for Smart Greenhouse Environment Management

**DOI:** 10.3390/s25196134

**Published:** 2025-10-03

**Authors:** Emmanuel Bicamumakuba, Md Nasim Reza, Hongbin Jin, Samsuzzaman, Kyu-Ho Lee, Sun-Ok Chung

**Affiliations:** 1 Department of Agricultural Machinery Engineering, Graduate School, Chungnam National University, Daejeon 34134, Republic of Korea; ebicamu@o.cnu.ac.kr (E.B.); reza5575@cnu.ac.kr (M.N.R.); samsuzzaman@o.cnu.ac.kr (S.); elkyu0927@cnu.ac.kr (K.-H.L.); 2 Department of Smart Agricultural Systems, Graduate School, Chungnam National University, Daejeon 34134, Republic of Korea; jhb0117@o.cnu.ac.kr

**Keywords:** smart greenhouse, wireless sensor network, environmental monitoring, Kalman filtering, predictive control

## Abstract

Management of smart greenhouses represents a transformative advancement in precision agriculture, enabling sustainable intensification of food production through the integration of multi-sensor networks, intelligent control, and sophisticated data filtering techniques. Unlike conventional greenhouses that rely on manual monitoring, smart greenhouses combine environmental sensors, Internet of Things (IoT) platforms, and artificial intelligence (AI)-driven decision making to optimize microclimates, improve yields, and enhance resource efficiency. This review systematically investigates three key technological pillars, multi-sensor monitoring, intelligent control, and data filtering techniques, for smart greenhouse environment management. A structured literature screening of 114 peer-reviewed studies was conducted across major databases to ensure methodological rigor. The analysis compared sensor technologies such as temperature, humidity, carbon dioxide (CO_2_), light, and energy to evaluate the control strategies such as IoT-based automation, fuzzy logic, model predictive control, and reinforcement learning, along with filtering methods like time- and frequency-domain, Kalman, AI-based, and hybrid models. Major findings revealed that multi-sensor integration enhanced precision and resilience but faced changes in calibration and interoperability. Intelligent control improved energy and water efficiency yet required robust datasets and computational resources. Advanced filtering strengthens data integrity but raises concerns of scalability and computational cost. The distinct contribution of this review was an integrated synthesis by linking technical performance to implementation feasibility, highlighting pathways towards affordable, scalable, and resilient smart greenhouse systems.

## 1. Introduction

Smart greenhouses utilize advanced technologies such as IoT, AI, and automation to optimize growing conditions, integrating sensors and control systems to regulate environmental parameters such as temperature, humidity, light intensity, and soil moisture. These systems aim to enhance resource efficiency, crop yield, and sustainability, addressing challenges in modern agriculture [1,2]. These controlled parameters directly influence plant health, productivity, and the quality of produce. Environment management in a smart greenhouse involves continuous monitoring and regulation of key parameters to maintain an optimal micro-climate for plant growth [3,4]. While existing studies have explored sensors, control systems, and data processing individually, few reviews integrate these components into a comprehensive framework. The objective of this paper is to provide a unified overview of multi-sensor monitoring, intelligent control, and data filtering methodologies, highlighting both their synergies and their limitations. This approach establishes the research gap and sets the foundation for the detailed review presented in the following sections.

An effective smart greenhouse fundamentally depends on the capacity to sense, analyze, and respond to environmental conditions. This capability is enabled by the seamless integration of multi-sensor networks, intelligent control systems, and advanced data processing techniques [5]. Multi-sensor monitoring facilitates a network of diverse sensors that continuously measure key environmental parameters within the greenhouse. This enables real-time monitoring of environmental variables, addressing the limitations of individual sensors in detecting specific parameters and thereby providing a thorough and reliable understanding of the greenhouse environment [6]. Multi-sensor monitoring utilizes sensors to deliver precise measurements of temperature, humidity, carbon dioxide, soil moisture, and light intensity, facilitating prompt responses and redundancy [7]. In a study involving six strategically placed sensor units, the average temperature recorded between 23:00 and 24:00 over two weeks was approximately 20 °C. The measurements of relative humidity and CO_2_ levels demonstrate considerable advantages, including effective monitoring across a network, although challenges such as increased costs and the need for calibration maintenance persist [8]. Recent research indicates that sensors are being more frequently integrated into environmental management systems to enhance accuracy for temperature and humidity (T: ±0.5 °C and H: ±2%) monitoring. However, light intensity and CO_2_ sensors were notably absent, particularly in dynamic and diverse greenhouse environments [9].

Multi-sensor systems present significant challenges in obtaining high-quality data, primarily due to issues like noise, various forms of redundancy, and inconsistencies. These factors create substantial obstacles to effective real-time environmental monitoring [10,11]. Failures in sensors can lead to gaps in coverage and may fragment the network into several partitions, rendering numerous functional nodes unreachable. Additionally, power outages and connectivity disruptions can significantly impact data processing, leading to lost data and an increase in data volume [12]. The integration of multi-sensor data presents challenges due to the variety of sensor types, each with its calibration, accuracy, and measuring scale [13].

Intelligent control refers to the use of advanced computational techniques to analyze sensor data and make autonomous decisions regarding climate control systems such as heating, ventilation, air conditioning, lighting, and irrigation. These systems dynamically adjust environmental variables to maintain optimal conditions, improving both efficiency and responsiveness. Data processing in this context involves the transformation of raw sensor data into meaningful information through cleaning, aggregation, normalization, and analysis. Efficient data processing enables the identification of patterns, trends, and anomalies that are critical for predictive and prescriptive analytics. Since sensors are often subject to external interferences and calibration issues, filtering methods such as Kalman filters, moving average filters, and wavelet transforms help in refining the data for reliable interpretation and control actions.

Traditional methods such as data smoothing and manual sensor calibration often fall short when addressing challenges in multi-sensor systems, emphasizing the necessity for improved approaches [14]. The positioning of sensors, the transfer of data, power usage, and the integration of multiple sensor networks are critical factors for effective data transmission. A need for innovative data filtering algorithms to ensure accurate and dependable integration of multiple sensors in real-time applications, such as smart greenhouses [15]. Challenges such as sensor anomalies, noise, and missing data require appropriate filtering methods for resolution and management [16]. Kalman filters are utilized in real-time applications to evaluate sensor data, enhancing the efficiency of machine learning and adaptive filtering algorithms [17].

Challenges such as sensor calibration, maintenance, and interoperability remain significant concerns. Ensuring accuracy and consistency across a diverse sensor network is complex, particularly when dealing with varying environmental conditions and sensor types. Moreover, intelligent control algorithms require robust training data sets and computational resources, which may not always be readily available. Data processing and filtering also pose challenges related to real-time performance, data storage, and bandwidth. The vast amount of data generated by sensor networks needs to be processed quickly and efficiently to enable timely responses. This necessitates the development of high-performance computing infrastructure and optimized algorithms. The integration of these technologies also demands specialized skills and knowledge, posing a barrier for traditional farmers and greenhouse operators. Training and capacity building are essential to bridge this knowledge gap. Additionally, system reliability and fault tolerance are critical in ensuring uninterrupted operation, especially in high-stakes agricultural settings. Besides, the initial investment in smart greenhouse technology can be prohibitive, particularly for small and medium-sized enterprises. The cost of sensors, controllers, software, and maintenance can be substantial, limiting widespread adoption.

Given the transformative potential of smart greenhouses in addressing global food security and sustainability challenges, it is imperative to understand and address the complexities associated with their core technological components. Recent reviews, such as a review of intelligent greenhouse systems based on Internet of Things control technology, enhancing greenhouse efficiency for integrating IoT and reinforcement learning for optimized climate control, and intelligent agricultural greenhouse control based on Internet of Things and machine learning, have provided valuable insights. However, these works focus mainly on IoT connectivity or machine learning aspects in isolation. In contrast, this review integrates multi-sensor monitoring, intelligent control, data processing, and filtering into a single comparative framework. By highlighting cross-technology comparisons, long-term sensor stability, filtering accuracy, and communication protocol alignment, our work offers practical implementation guidance that distinguishes it from existing studies.

While numerous studies have explored individual aspects of smart greenhouse systems, there remains limited work that integrates multi-sensor monitoring, intelligent control, data processing, and filtering within a single comparative framework. This review article was motivated by the need to bridge this gap in the literature by providing a holistic overview of these interrelated technologies. By synthesizing recent advancements, identifying best practices, and highlighting prevailing challenges, this article aims to serve as a foundational reference for researchers, practitioners, and policymakers interested in smart greenhouse systems. Furthermore, as technology continues to evolve rapidly, it is crucial to continuously update the knowledge base to reflect current trends, innovations, and future directions. This review aims to contribute to this dynamic field by offering an update and in-depth analysis of the state of the art. The objective of this paper was to provide a review of the technologies for smart greenhouse environment management, with a specific focus on multi-sensor monitoring, intelligent control systems, data processing and filtering techniques.

## 2. Methods

A systematic literature review was conducted in accordance with the PRISMA guidelines to identify relevant studies on smart greenhouse systems. The review process began with the identification of 1500 studies from major academic databases, including IEEE Xplore, ScienceDirect, Springer, MDPI, and Web of Science, using a combination of keywords such as smart greenhouse, sensor networks, intelligent control, data filtering and artificial intelligence. An additional 200 studies were removed at this stage due to clear lack of relevance to the scope of this review study.

During the screening phase, a total of 1300 studies were screened based on their titles and abstracts. From these, 300 articles were identified for full-text retrieval, of which 250 articles were successfully accessed and assessed for eligibility. Following the application of predefined inclusion and exclusion criteria, 136 studies were removed: 70 due to insufficient relevance to the greenhouse systems, 40 due to methodological weaknesses, and 26 for being published in languages other than English.

**Figure 1:**
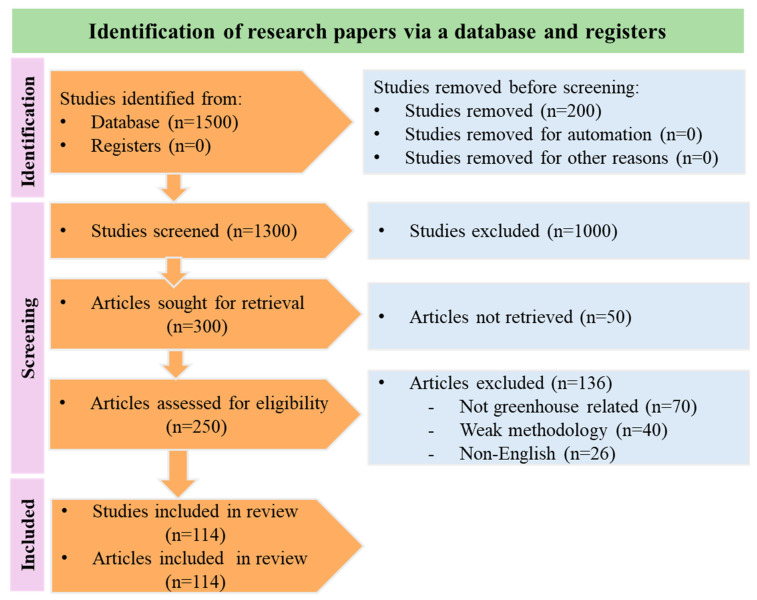
PRISMA flow diagram of the systematic literature review on smart greenhouse technologies, illustrating study identification, screening, eligibility assessment, and final inclusion from key databases between 2010 and 2025.

Ultimately, 114 articles met the eligibility criteria and were included in the final review. The overall selection process, including the number of studies identified, screened, excluded, and retained, is illustrated in the PRISMA style flow diagram as shown in Figure 1, which provides visual details of the study identification, screening, and inclusion process.

The gathered information was organized into three primary areas, including multi-sensor environmental monitoring, intelligent control strategies, and data processing and filtering methodologies to enable systematic data synthesis. The field of multi-sensor environmental monitoring includes sensor technologies for measuring temperature, humidity, CO_2_ concentration, light intensity, and energy management. The examination of control strategies included model predictive control, reinforcement learning, AI-driven heating, ventilation, and air conditioning (HVAC) optimization, and IoT-based actuation mechanisms. The review focused on data filtering methodologies, specifically Kalman filtering, neural network-based models, and hybrid filtering techniques. Each domain underwent a thorough examination grounded in essential performance metrics, such as sensor accuracy, latency, computational complexity, scalability, and the feasibility of integration within smart greenhouse systems. The assessment of multi-sensor monitoring systems considers essential factors, including sensor placement, calibration methods, sensor fusion strategies, and wireless communication technologies. The greatest focus was placed on how the arrangement of spatial sensors and the calibration methods influence the precision of measurements.

**Figure 2:**
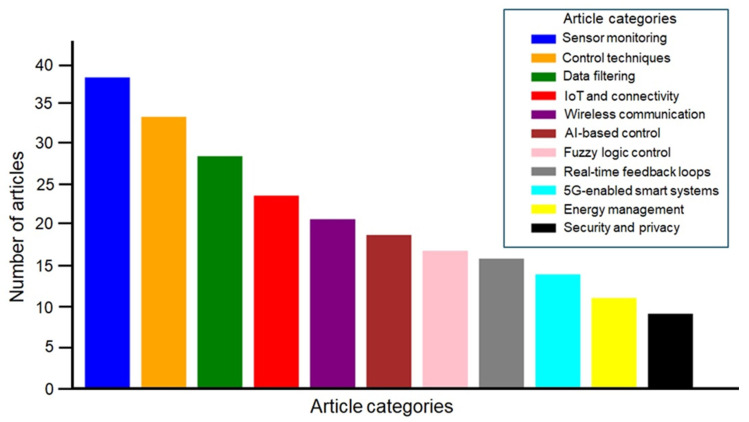
Distribution of reviewed articles by key technological categories in smart greenhouse research, highlighting major focus areas and comparatively underexplored domains during 2010–2025.

The frameworks for data integration were evaluated to improve measurement reliability and reduce redundancy. An analysis was conducted on wireless communication protocols such as ZigBee, LoRa, Wi-Fi, and NB-IoT, focusing on their range, power efficiency, and data transmission performance within greenhouse environments. Figure 2 illustrates the distribution of reviewed articles across different technological categories within smart greenhouse research. The analysis shows that sensor monitoring and control techniques constitute the most prominent research hotspots, reflecting the central role of environmental sensing and automation in advancing greenhouse technologies. Data filtering and IoT and connectivity also emerge as well-studied areas, highlighting the importance of data-driven decision-making and integrated communication networks in modern greenhouse systems. Although this is a presentation of aggregate distributions, a closer inspection of publication years reveals that research on AI-based control, 5G-enabled systems, and security has gained momentum mainly after 2020, reflecting their status as emerging trends. Regional variations are also evident, with Asian countries contributing more extensively to IoT, sensor monitoring, and AI-based greenhouse applications, while European research shows greater emphasis on energy management and sustainable control systems.

To tackle issues in real-time data processing, data filtering approaches were methodically assessed for their efficacy in noise reduction, missing data imputation, and anomaly identification. The performance of time-domain filters, including moving average and low-pass filters, was evaluated for signal smoothing. Frequency-domain methods, such as band-pass filtering, were evaluated for their effectiveness in reducing high-frequency noise. Statistical filtering methods, specifically Kalman filters, were examined for real-time sensor data integration and outlier rectification. AI-driven filtering models, such as neural networks and deep learning approaches, were examined for their adaptability to diverse sensor datasets and fluctuating ambient circumstances. The efficacy of various filtering methods was evaluated based on accuracy, computational economy, and flexibility for multi-sensor integration. An extensive evaluation of control systems for regulating greenhouse environments was carried out, highlighting optimization frameworks powered by artificial intelligence for managing heating, ventilation, air conditioning, and humidity. The study examined predictive and adaptive control algorithms and their impact on power and energy efficiency. Various control methodologies, including fuzzy logic, genetic algorithms, particle swarm optimization, and machine learning-based controllers, were assessed for their ability to improve environmental stability and decrease energy consumption. Additionally, systems for managing energy that combine thermal and electrical load-balancing strategies were assessed to enhance overall efficiency and sustainability.

This review recognizes limitations, including diverse data sources, the distinction between simulation and real-world implementation, and constraints related to computational resources. The inconsistency in data reporting among various studies presented a significant challenge. These inconsistencies arise from varied calibration standards, simulation versus field data discrepancies, and inconsistent reporting of metrics such as accuracy, latency, and energy consumption. To mitigate this, we cross-validated data with benchmark standards, prioritized studies with both simulation and field validation, and recommended that future greenhouse research adopt minimum reporting datasets to enhance reproducibility and comparability. Several examined methods were evaluated in controlled environments instead of actual greenhouse conditions. Implementing AI-driven control models within low-power IoT frameworks continues to pose significant challenges. Ethical approval was necessary for this study, given that it is a literature-based review that does not involve human or animal subjects. All data sources are accessible to the public, and the referenced materials are cited correctly. The information underpinning this review comes from publicly accessible sources, and all studies referenced have been properly cited. Further details necessary for replication are available upon request.

## 3. Results

This section synthesizes key findings from the reviewed literature, focusing on the integration of multi-sensor monitoring, intelligent control, and advanced data processing and filtering techniques in smart greenhouse environment management. Smart greenhouse systems rely heavily on multi-sensor architectures to monitor a range of environmental parameters, including temperature, relative humidity, CO_2_ concentration, solar radiation, soil moisture, and energy consumption. The use of diverse sensing technologies—such as electrochemical, optical, capacitive, and MEMS-based sensors—enables high-resolution, real-time data acquisition. These sensors are strategically deployed to capture spatial and temporal microclimate variations within greenhouse zones, which is essential for precision control of growing conditions. Effective data transmission is facilitated by wireless communication protocols, including ZigBee, LoRaWAN, Wi-Fi, and Bluetooth Low Energy (BLE). These technologies ensure low-power, long-range, and reliable communication between distributed sensor nodes and centralized control systems. The integration of Internet of Things (IoT) platforms further enables seamless data flow and real-time monitoring. Advanced architecture combining edge computing and cloud infrastructure allows localized decision-making while supporting centralized data analytics and long-term storage.

Intelligent environmental regulation is achieved through the application of AI-based control algorithms, including model predictive control (MPC), fuzzy logic control, and reinforcement learning frameworks. These techniques enable adaptive and predictive management of actuators (e.g., fans, vents, heaters, misters), maintaining optimal growth conditions while minimizing energy and water consumption. The integration of control models with real-time sensor feedback improves the robustness and responsiveness of the system. To ensure accurate and reliable sensor data, various signal processing and filtering techniques are employed. Approaches such as Kalman filtering, Savitzky–Golay smoothing, and adaptive thresholding help mitigate sensor noise and transient fluctuations. More advanced methods, including neural-network-based filtering and sensor fusion algorithms, support improved data integrity and robustness in dynamic greenhouse environments. These techniques are particularly useful for managing sensor drift, data loss, and environmental disturbances.

AI-enabled anomaly detection techniques, such as autoencoders, statistical learning models, and hybrid rule-based systems, are increasingly used to identify faults in sensor readings or actuator performance. These methods enhance the reliability of greenhouse operations by enabling early intervention, predictive maintenance, and reducing system downtime. The combined use of multi-sensor networks, intelligent control systems, and robust data processing frameworks significantly enhances greenhouse sustainability. These technologies contribute to improved climate control precision, optimized resource utilization, and reduced operational costs, while supporting adaptive and autonomous decision-making. Collectively, they represent a significant advancement in the transition toward data-driven, resilient, and resource-efficient greenhouse farming.

### 3.1. Multi-Sensor Monitoring in Smart Greenhouse Management

Multi-sensor monitoring refers to the use of multiple, complementary sensors within a greenhouse facility to simultaneously capture key environmental variables, such as air temperature, relative humidity, soil moisture, light intensity, CO_2_ concentration, and energy consumption. The core idea is to create a comprehensive, high-resolution snapshot of the greenhouse microclimate, enabling more precise control and early detection of anomalies that could impact plant health or productivity [18]. Monitoring through environmental sensors in smart greenhouses is essential for enhancing growing conditions and ensuring sustainability. Different types of sensors were used to monitor environmental parameters inside and outside greenhouses. Temperature sensors provide real-time data that helps maintain the appropriate thermal conditions for plant growth, thereby enhancing yield and energy efficiency [19,20]. Common types include thermistors—ceramic-based sensors with high sensitivity over a limited range, and resistance temperature detectors (RTDs), typically platinum-based, which offer accurate, linear readings over a wider range. Humidity sensors play a vital role in regulating moisture levels and ensuring that plants do not suffer from dehydration or excessive moisture, both of which can result in fungal diseases [21]. These typically use capacitive or resistive elements with hygroscopic materials that alter capacitance or resistance based on ambient humidity, enabling accurate relative humidity measurement. CO_2_ sensors, such as non-dispersive infrared (NDIR) types, monitor carbon dioxide levels to support photosynthesis and enable CO_2_ enrichment [22,23,24]. These sensors measure the absorption of infrared light by CO_2_ molecules, correlating it with concentration levels. Light sensors ensure that plants get enough light, minimizing the need for artificial lighting and saving energy [25,26]. They include photodiodes, phototransistors, and light-dependent resistors (LDRs), which change resistance or current with light intensity. Advanced options like photosynthetically active radiation (PAR) sensors measure light in the 400–700 nm range, crucial for photosynthesis, enabling smart lighting control. Energy sensors track power consumption, facilitating the efficient use of electricity and other resources, thereby enhancing the system’s sustainability [27]. This Hall-effect sensor detects magnetic fields from current flow and converts them into proportional voltage outputs. It enables real-time monitoring, fault detection, and energy optimization. These sensors, when deployed collectively in a multi-sensor system, enhance crop yield and resource efficiency while fostering sustainable agricultural practices by reducing water consumption and minimizing the environmental impact of greenhouse operations.

Recent studies indicated that incorporating sensor networks in smart greenhouses can result in 70% model training and 30% for testing to reduce water use [28,29] and around 65–85% of total energy consumed by heating greenhouses for energy efficiency [30], positioning them as a crucial element of modern precision agriculture. Wardani et al. [31] developed a cost-effective sensor network for temperature and humidity monitoring using front, middle, and rear sensor placements, achieving high accuracy (RMSE: 1.48 °C, 3.18% RH; R: 0.81–0.85). However, environmental variability and long-term sensors pose calibration challenges. Prskalo et al. [32] proposed a modular control system integrating temperature, humidity, soil moisture, and light sensors to regulate ventilation, irrigation, lighting, and heating with crop-specific optimization via a touchscreen interface. Another study [33] introduced a LoRa-based environmental monitoring network for vegetable greenhouses, offering reliable real-time data transmission with low packet loss (<0.05%) and cost-effective coverage, while highlighting spatial variability across microclimate variables such as temperature (1.6 °C) and CO_2_ (141 ppm).

Chodorek et al. [34] advanced aerial sensing with a flying weather station, emphasizing reliable data transmission under congested networks. Complementarily, integrated video-sensor systems [35] faced video latency issues during real-time greenhouse monitoring. Ground-deployed sensor arrays [36] revealed humidity sensor interference due to dew and soil moisture sensor limitations from inconsistent soil contact. Soheli et al. [37] implemented an ANFIS-IoT system for winter crop optimization, combining fuzzy logic and neural prediction with a 93.62% attack detection rate in the IoT perception layer. A handheld wireless system [38] provided real-time environmental feedback with a 200 m range and high accuracy, enhancing portability and usability. Collado et al. [39] designed an IoT-based agro-climatic monitoring system with internal/external sensor stations transmitting data via Wi-Fi to a cloud platform, addressing challenges like Wi-Fi coverage and secure access in large-scale melon production [40].

Additional efforts focused on predictive climate control and emissions tracking. Hernandez-Morales et al. [41] demonstrated accurate 24-h climate forecasting using spatial sensor deployment strategies, while a parallel study [42] applied embedded systems to monitor CO_2_ emissions from soil respiration. Cloud-based monitoring approaches [43] expanded applications to atmospheric pollution tracking using MQTT-enabled multi-sensor arrays. Astutik et al. [44] developed an Android-integrated system for hydroponic greenhouses, monitoring temperature, humidity, light, and nutrient levels with low error rates. Similarly, Ittaqullah et al. [45] introduced a fuzzy logic-based temperature and light control system with a minimal error of 0.18% between simulation and field data, confirming reliability. A system, comprising 20 LoRaWAN-enabled sensor nodes deployed [46] at two vertical levels, monitored environmental conditions in real-time and projected sensor data into a normalized scale (0–1) representing deviation from optimal crop-specific thresholds. Mean sensor values showed poor environmental regulation during warmer periods. This study reveals that light conditions, greenhouse geometry, and HVAC placement significantly affect microclimate uniformity.

A compact electrical monitoring system recorded voltage, current, and temperature at one-minute intervals, both locally and via remote platforms, and was validated against a high-precision power analyzer (±0.03% accuracy) [47,48]. In greenhouse environments, spatial monitoring was conducted using distributed sensor networks measuring temperature, humidity, CO_2_ concentration, light intensity, and soil moisture to assess microclimate variability [49]. Additional systems integrated sensors for electrical conductivity and fertilizer pH, achieving a 23.6% reduction in grid energy use, thereby demonstrating the impact of renewable energy adoption on sustainable greenhouse operations [50]. For low-level power consumption measurement, current sensors and microcontrollers were employed, with high linearity (R^2^ = 0.99) and precision. Measurement errors were limited to 2.3% for voltage, 7% for current, and 5.8% for power, while coefficients of variation remained below 0.4%, confirming the system’s reliability for accurate electrical monitoring in precision agriculture applications [51]. Table 1 presents a comparison of environmental sensors utilized for real-time monitoring in smart greenhouses.

Collectively, these innovations illustrate the shift towards cost-efficient, scalable, and reliable multi-sensor systems that enhance spatial-temporal resolution, predictive analytics, and automated control in precision greenhouse agriculture. At the same time, the development of sensor technology has evolved from early single-purpose devices to multifunctional, IoT-enabled systems. Table 1 shows groups of sensors into categories such as temperature, humidity, carbon dioxide, light, and energy. Their parameters, such as accuracy, power consumption, and transmission distance, directly determine suitability in different greenhouse conditions. Low-power sensors suit battery-driven IoT nodes, whereas High-accuracy carbon dioxide sensors are essential for large commercial greenhouses with enrichment systems.

**Figure 3:**
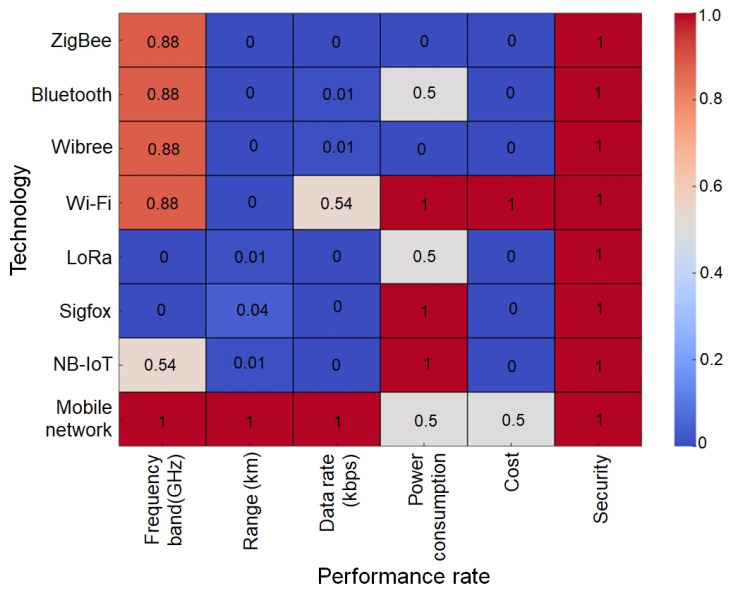
Comparative analysis of wireless communication technologies for smart greenhouse monitoring across frequency band, range, data rate, power consumption, cost, and security (note: security is shown uniformly in the heatmap, though in practice it varies considerably across technologies).

The choice of wireless protocol depends strongly on greenhouse size, structure, and crop conditions. LoRa is suited for large-scale greenhouses with dense canopies because of its long transmission range and robustness, despite low data rates. ZigBee is effective for smaller greenhouses that require dense, low-power sensor networks. Wi-Fi provides higher bandwidth but consumes more energy, making it appropriate only for mains-powered systems or scenarios demanding high data throughput [52]. The comparative analysis of wireless communication technologies for WSNs compares eight wireless communication technologies using five key performance indicators, such as frequency band, range, data rate, power consumption, cost, and security. Technologies are evaluated using a scale ranging from 0 to 1, where 1 indicates the peak performance and 0 signifies the minimum performance in each category [53,54]. Recent studies indicate that ZigBee, Bluetooth, Wibree, and Wi-Fi all operate within the 2.4 GHz ISM band, offering a suitable range and susceptibility to interference. Mobile Networks lead in coverage, offering extensive reach, while LoRa and Sigfox are more appropriate for long-range applications [55,56,57]. Wi-Fi, mobile networks, and NB-IoT provide optimal data rates, making them well-suited for applications requiring high bandwidth. Zigbee, Bluetooth, and Wibree were developed to facilitate communication with low data rates while prioritizing energy efficiency. ZigBee, Bluetooth, and Sigfox demonstrate cost-effectiveness due to their streamlined infrastructures, rendering them ideal for both consumer-grade applications and extensive sensor networks [58]. Regarding security, various technologies such as ZigBee, Wi-Fi, NB-IoT, and Mobile Networks provide strong encryption, guaranteeing secure communication and data transmission [59,60]. Figure 3 presents a comparative evaluation of wireless communication technologies commonly applied in smart greenhouse systems, on a relative scale (0–1) to highlight the understanding. The results indicate that ZigBee and Bluetooth are cost-effective and energy-efficient, making them suitable for dense in-house sensor networks, though limited by short range and modest data rates. LoRa and Sigfox extend communication across kilometers but offer very low throughput, restricting use to telemetry. Wi-Fi and cellular/NB-IoT enable higher data rates and broader coverage, supporting advanced tasks such as video monitoring and cloud integration, but at higher energy and financial costs. Security levels vary, with cellular and modern Wi-Fi offering stronger encryption, while ZigBee, Bluetooth, LoRa, and Sigfox provide weaker or implementation-dependent protection. While the heatmap normalizes all dimensions on a 0–1 scale, it should be noted that the depiction of security as uniform across technologies is a limitation of the visualization rather than a reflection of actual capabilities.

**Figure 4:**
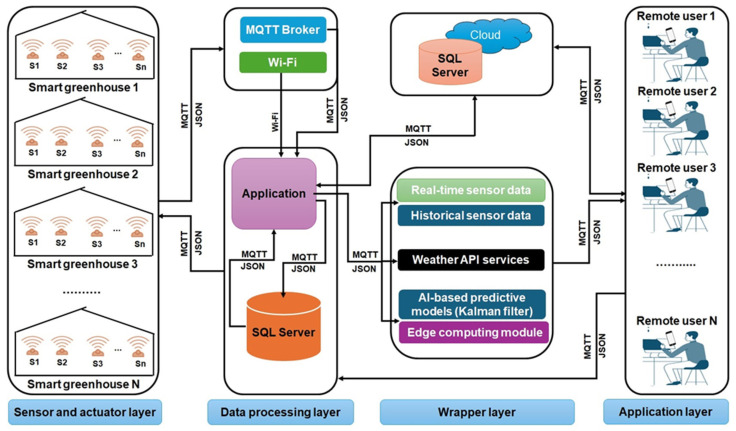
A general architecture of multi-sensor network-based monitoring of a smart greenhouse.

Real-time multi-sensor data fusion is a critical challenge in smart greenhouse monitoring, requiring robust solutions to address noise, data inconsistencies, and heterogeneity. Advanced techniques like Kalman filtering and wavelet transform significantly minimize measurement errors while maintaining critical data trends, thereby enhancing data reliability and the accuracy of decision-making [61]. The integration of multi-sensor data is crucial for improving environmental control systems. The implemented fusion algorithm successfully minimized temperature fluctuations based on greenhouse temperature monitoring. There are two main reasons for the high error in monitoring results the poor quality of data collected by wireless sensor networks and the low accuracy of fusion algorithms. The measuring error of the temperature sensor is generally about ±0.5 °C, and that of the sensor with higher accuracy is about ±0.3 °C [62,63]. Furthermore, the precision of humidity control saw an improvement of 15% while water usage efficiency rose by 33%, demonstrating the benefits of integrated data processing techniques [64]. The process of making decisions in real time is enhanced by machine learning models that derive actionable insights from ongoing data streams. This review study demonstrates that the integration of machine learning with multi-sensor data improved pest detection accuracy, reaching 81% and decreased response times to soil and environmental data by 95.9% [65,66]. Figure 4 illustrates a multi-layered architecture that combines diverse sensors and data fusion methods for real-time environmental monitoring. This innovative and flexible system enhances plant growth, maximizes resource efficiency, and improves greenhouse management.

### 3.2. Intelligent Control Techniques in Smart Greenhouse Management

Intelligent control systems have transformed the management of smart greenhouses through the integration of IoT, cloud computing, and AI-based decision-making. Traditional manual methods, though cost-effective, lack consistency, while automated approaches leveraging IoT sensors and cloud-based analytics have significantly improved scalability, accuracy, and energy efficiency [67]. Modern greenhouse automation extends beyond conventional systems by incorporating adaptive intelligence, such as fuzzy logic, rule-based automation, and AI-powered real-time feedback loops. Combining IoT-enabled sensors with cloud computing improves real-time environmental monitoring and control. Wireless sensor networks (WSNs) continually gather and send microclimate data (temperature, humidity, CO_2_ concentration, and light intensity to cloud-based platforms, where predictive algorithms maximize greenhouse operations [68]. AI models trained on historical and real-time data dynamically adjust climate parameters to maintain optimal conditions. AI-driven irrigation scheduling in tomato greenhouses reduced water use by about 60% in multi-season trials using real-time soil moisture and climate feedback. The same study showed that weed management in lettuce greenhouses caused yield losses of 48–60% under controlled temperature by 24 ± 2 °C and humidity by 60–70%, depending on the intervention strategy [69].

A hybrid control approach that integrates fuzzy logic with rule-based automation offers strong decision–making capabilities in uncertain environments. Fuzzy logic controllers analyze vague data from IoT sensors to facilitate seamless transitions between various environmental states [70]. Instead of strict on/off temperature changes, fuzzy logic allows for gradual modulation of heating and cooling systems by responding to real-time deviations from set points. Rule-based automation enhances operational efficiency by establishing logical conditions. Should CO_2_ levels fall below 350 ppm and sunlight exceed 300 W/m^2^, initiate CO_2_ injection to minimize excess energy use while promoting ideal conditions for plant growth [71]. Real-time feedback loops driven by AI improve accuracy and flexibility in intelligent greenhouses. Through the application of deep reinforcement learning (DRL) and model predictive control (MPC), the system persistently adapts to environmental variations and responds effectively to control reactions. This optimization, powered by AI, effectively reduces the energy cost associated with HVAC systems.

(1) $$J=\alpha \cdot (T_{error}^{2}+H_{error}^{2})+\beta \cdot E_{HVAC}$$

In this formulation, the total control objective J balances environmental stability with energy efficiency. The weight coefficients α and β were determined through iterative simulations using historical greenhouse data, with convergence typically achieved after 150–2900 training episodes. Features variables included both environmental conditions, such as temperature, humidity, carbon dioxide, and light, for crop growth stage parameters, since leafy vegetables and fruiting crops often require different priorities. For instance, α emphasized for lettuce to maintain strict humidity control, while β is prioritized for melons to reduce HVAC energy consumption. This adaptive strategy improves reproducibility and ensures the control model remains practical across multiple crop types. As a result, the system not only stabilizes the microclimate inside the greenhouse but also reduces operating costs by continuously optimizing the trade-off between precision control and energy efficiency [72,73]. Reinforcement learning algorithms systematically modify HVAC parameters by utilizing real-time sensor feedback, thereby achieving adaptive and energy-efficient climate control.

**Figure 5:**
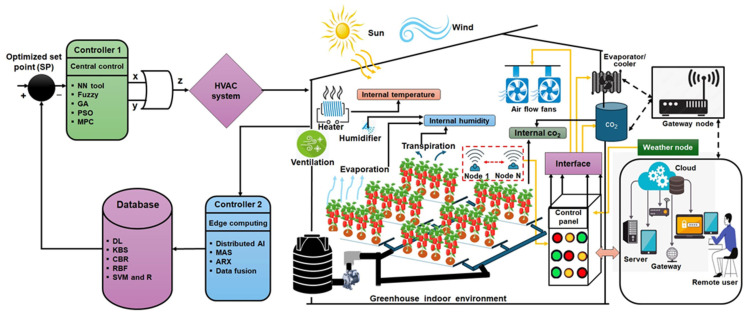
Intelligent control system for smart greenhouse management.

To enhance system intelligence, edge computing complements cloud processing by enabling localized decision-making, reducing latency, and ensuring operational continuity in the event of network disruptions. The predictive control architecture, depicted in Figure 5, features a multi-layer structure that includes an edge layer (controller 2) designed to process real-time data locally through AI models (multi-agent systems, ARX, and data fusion) for prompt control actions [74]. The cloud layer (controller 1) implements advanced algorithms, including neural networks, fuzzy logic, genetic algorithms, PSO, and MPC for global optimization and long-term planning [75]. The database layer is responsible for storing and analyzing historical data through the application of deep learning (DL), case-based reasoning (CBR), and support vector machines (SVM) to facilitate predictive decision-making [76]. This multi-layered approach ensures robust, real-time adaptation to environmental fluctuations while optimizing energy efficiency. The IoT-cloud synergy further facilitates remote monitoring and control, allowing farmers to manage greenhouse conditions via mobile applications [77]. While methods such as fuzzy logic, reinforcement learning, and model predictive control each offer clear benefits, and constraints in real-world use. Fuzzy logic requires constraint fine-tuning, reinforcement learning depends on large, clean datasets and struggles with noisy inputs, and MPC, though accurate, was often too computationally heavy for small-scale [78,79,80]. To give the readers a practical reference, we expanded Table 2 to present a side-by-side comparison of these approaches, outlining both their strengths and the challenges of applying them in greenhouse management.

Intelligent control techniques optimize greenhouse environments through multi-sensor data integration, but challenges persist. Calibration challenges and inconsistencies in data affect reliability, necessitating regular recalibration. Handling extensive data sets requires significant computational resources, and adaptive algorithms often face challenges due to intricate environmental variations [81]. The significant energy consumption observed in actuator systems calls for predictive optimization, which could potentially lead to a 23.8% reduction in usage [82]. Nonetheless, challenges related to interoperability stemming from proprietary technologies obstruct smooth integration, while cybersecurity risks present significant threats to system security [83]. Economic limitations hinder adoption, especially among small-scale farmers, as they face significant initial investment costs. Moreover, erratic environmental factors necessitate strong predictive models for resilience [84,85]. Progress in artificial intelligence, sensor technology, and energy-efficient solutions is essential for addressing these challenges and achieving sustainable management of smart greenhouses. Table 2 summarizes the key intelligent control techniques applied in smart greenhouse management, highlighting their core functionalities, parameters addressed, and practical applications within greenhouse environments. Table 2 shows the structure in layers, such as data acquisition, dynamic control, and optimization. Each technique is linked to specific greenhouse functions, such as model predictive control for HVAC optimization. This layered presentation clarifies applicability boundaries and collaborative logic.

**Table 2:** Comparative overview of intelligent control techniques in smart greenhouse management.

Technology/Method	Key Parameters	Advantages	Limitations	Applications	Ref.
IoT-based monitoring	Temperature, humidity, CO_2_, light for real time monitoring	Monitoring and scalable	Network stability is not stable	Greenhouse monitoring	[67,68,69]
Fuzzy control logic	HVAC modulation	Smooth transitions and handle vague data	Limited prediction	An environment with frequent fluctuations	[70]
Rule-based automation	Threshold-based triggers	Transparent and low cost	Not predictive and lacks adaptability	Irrigation and carbon dioxide control	[71]
Model predictive control	Forecast-based climate regulation	Optimize energy and predictive accuracy	Computationally demanding	Large and commercial greenhouse	[72,73]
Reinforcement learning	Adaptive HVAC and anomaly detection	Highly adaptable	Require large datasets	Complex and dynamic environment	[72,73]
Edge cloud hybrid	Local real-time and global optimization	Low latency, resilient to disconnect	High setup complexity	Networked smart greenhouse systems	[74,75,76]
Mobile/remote IoT control	Remote access and alerts	Convenient and user-friendly	Requires strong connectivity	Farmer-oriented monitoring, quick response	[77]
Predictive analytics and fusion	Historical trend and variability analysis	Long-term insights	Sensitive to data quality	Climate forecasting and decision support	[76,84,85]

### 3.3. Data Processing and Filtering for Smart Greenhouse Management

**Figure 6:**
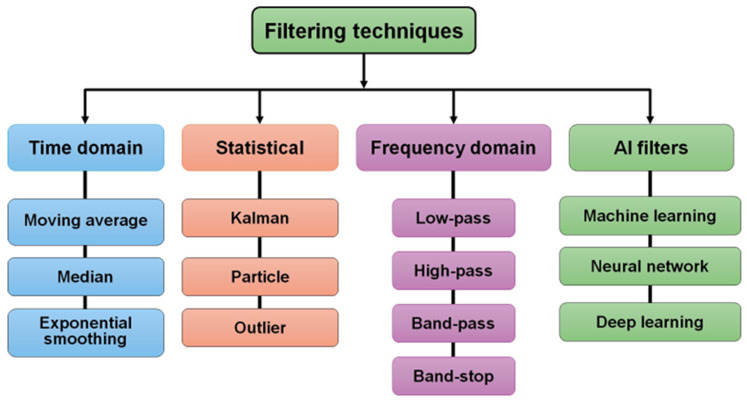
Classification of data filtering methods for environmental monitoring systems.

Efficient data processing and filtering are essential for multi-sensor networks in smart greenhouse management. This ensures high-quality signal capture, minimizes noise interference, addresses missing data, and enhances system reliability. The intricate nature of sensor networks and IoT-driven environmental monitoring systems necessitates the development of enhanced filtering algorithms, which are vital for facilitating real-time decision-making and optimizing climate control. Data filtering methods, including time-domain, frequency-domain, statistical, and AI-driven approaches, offer distinct advantages for addressing a range of challenges in sensor-based monitoring [86]. Techniques such as moving average, exponential smoothing, and median filters are effective in minimizing noise and enhancing signal stability. A moving average filter decreases temperature noise, enhancing the management of greenhouse climates [87]. In scenarios characterized by significant interference, employing frequency-domain techniques such as low-pass, high-pass, band-pass, and band-stop filters can effectively separate preferred signals from high-frequency noise, enhancing signal precision by testing results were impressive, showing that the filter measured center frequency could be tuned across a wide spectrum, from 1.1 to 3.1 GHz, achieving a relative bandwidth of up to 95% [88]. Kalman and particle filters provide dynamic data fusion, enable anomaly detection, and facilitate predictive corrections, thereby enhancing the reliability of sensor data. Recent findings indicate that Kalman filters enhanced the accuracy of precision agriculture data [89]. AI-powered filtering techniques driven by artificial intelligence, leveraging machine learning and deep neural networks, have revolutionized the processes of anomaly detection, fault diagnosis, and predictive analytics. A deep learning-based filter in IoT-enabled greenhouse monitoring systems identified 87% of faults, showcasing its capability for real-time predictive maintenance and decision support [90,91]. Edge computing and cloud-based analytics improve sensor network efficiency by lowering latency and bandwidth. These technologies increase data completeness, integrate disparate sensor inputs, and identify abnormalities for resilient system performance. Figure 6 illustrates how environmental monitoring systems filter data for real-time greenhouse management. IoT-driven smart greenhouse systems require advanced data filtering and processing for effective temperature management, resource efficiency, and agricultural production.

**Figure 7:**
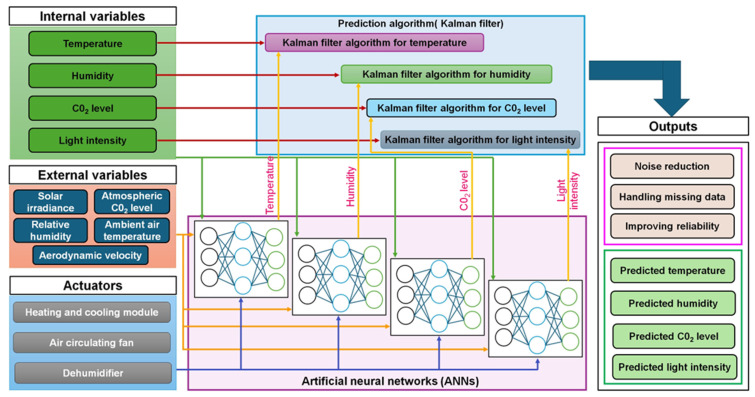
Integrated Kalman filter and neural network architecture for environmental parameter prediction.

The Kalman filter and neural network framework for multi-sensor data, prediction algorithms, and external environmental factors play a crucial role in ensuring reliable system operation. Figure 6 demonstrates the monitoring of essential environmental parameters, including temperature, humidity, CO_2_ levels, and light intensity, achieved through sensors and processed with a blend of Kalman filters and artificial neural networks (ANNs) [92]. The Kalman filter is well-known for its ability to handle noisy and incomplete data, facilitating precise predictions by integrating sensor inputs with external factors such as solar irradiance, ambient air temperature, and atmospheric CO_2_ levels [93]. ANNs improve traditional filtering by simulating complicated, nonlinear input-output interactions to improve prediction skills. The hybrid technique lowers noise, adjusts for missing data, and increases forecast reliability, enabling precise temperature management using actuators like heating and cooling modules, fans, and dehumidifiers [94]. Incorporating external factors allows for greater flexibility in response to changing outdoor conditions, thereby improving the precision of internal climate regulation. Recent studies show that these integrated systems can lead to ensemble hybrid-based intrinsic time-scale decomposition, which has reduced the root mean square by 24% for Klang and 34% for Langat, respectively, compared with the intrinsic time-scale decomposition-conventional neural network model [95,96,97]. The advancements lead to optimized energy consumption, improved crop yields, and more sustainable greenhouse operations, underscoring the potential for further integration of AI and IoT technologies. A comparative view of filtering methods highlights important trade-offs. The Kalman filter was highly efficient for real-time noise correction but less effective for nonlinear processes. Frequency domain filters are lightweight and effective under stable conditions but lack adaptability to dynamic environments. AI-based approaches, including neural network filters, offer superior adaptability and fault detection, though they require greater computational and energy resources. This cross-comparison provides practical guidance for selecting the most suitable filtering techniques in different greenhouse scenarios. As summarized in Table 3, these findings illustrate how filtering approaches can be categorized into traditional, statistical, AI-driven, and hybrid methods, each with distinct advantages and limitations. Traditional filters such as moving averages and frequency-domain methods remain useful for basic noise suppression under stable conditions, while statistical models like the Kalman and particle filters offer robustness in sensor fusion and anomaly detection but are constrained by assumptions and computational demands. In contrast, AI-powered and hybrid techniques (e.g., Kalman–ANN, ensemble models) demonstrate greater adaptability and predictive capability in complex and nonlinear greenhouse environments, though they require more data and processing resources as shown in Figure 7. Edge- and cloud-based strategies further enhance scalability and resilience by enabling real-time distributed processing.

The challenges encountered in real-time data processing and filtering techniques encompass scalability, computational limitations, and the variability of environmental conditions. Extensive multi-sensor networks produce enormous amounts of data, putting significant pressure on processing resources, particularly in edge computing and remote applications where power is limited [98]. Variable noise levels, sensor instability, and sudden shifts in the environment necessitate the implementation of adaptive filtering techniques, while challenges related to sensor reliability and energy efficiency add complexity to real-time operations [99]. To tackle these challenges, future developments should concentrate on lightweight, adaptive filtering algorithms that enhance accuracy while minimizing computational overhead. Hybrid filtering approaches that combine AI with statistical methods can enhance adaptability while ensuring efficiency is preserved [100]. The integration of edge AI and distributed processing is set to improve real-time decision-making by reducing latency. Additionally, self-learning algorithms will be capable of dynamically modifying filtering parameters in response to changes in the environment [101]. Furthermore, creating energy-efficient AI models and utilizing hardware acceleration will be crucial for enhancing processing power in resource-constrained environments. Improving multi-sensor fusion methods will significantly enhance data reliability and reduce noise, leading to more robust filtering techniques for applications in smart agriculture, UAV-based monitoring, and precision irrigation.

## 4. Discussion

Modern technologies like IoT, edge computing, and AI are transforming agricultural monitoring and control systems. The recent developments emphasize the importance of real-time data collection, sophisticated control strategies, and effective data filtering and processing to enhance decision-making and resource utilization [102]. The analysis of multi-sensor monitoring, intelligent control techniques, and data filtering methods underscores the significant influence of these technologies on enhancing agricultural efficiency and productivity [103]. The implementation of multi-sensor monitoring has greatly enhanced the process of acquiring agricultural data by allowing for the simultaneous measurement of essential environmental parameters such as soil moisture, temperature, and nutrient levels. The development of self-calibrating and multi-functional sensors has improved measurement precision while lowering operational expenses. Investigations reveal that implementing these sensors in regulated settings led to a reduction in operational costs and an enhancement in data precision [104]. Moreover, sensor networks empowered by IoT facilitate ongoing monitoring and instantaneous data transmission, addressing the difficulties linked to conventional discrete measurement systems.

Intelligent control strategies are essential for enhancing agricultural practices, especially in the areas of precision irrigation and environmental management. The implementation of AI-driven control frameworks facilitates dynamic decision-making informed by real-time sensor data. Edge computing architectures enable decentralized control operations, minimizing latency and enhancing system responsiveness. Findings indicate that intelligent control methods have led to a reduction in irrigation decision-making time and enhanced water use efficiency [105]. Furthermore, strong control algorithms facilitate scalability across diverse agricultural environments; however, there are still obstacles in tailoring these algorithms to different crop varieties and environmental factors. Effective AI-based filtering can ensure data integrity through specific approaches such as hybrid Kalman-ANN models, Bayesian filtering frameworks, and attention-based deep learning models. These approaches improve accuracy by handling noise, uncertainty, and heterogeneous datasets, suggesting that they improve data reliability [106]. Future development should prioritize lightweight edge AI models to operate on low-power hardware, and explainable AI frameworks to enhance transparency and farmer adoption.

The assessment of multi-sensor monitoring and intelligent control systems highlights their benefits as well as the challenges they present. Most reported advances in multi-sensor monitoring and intelligent control remain validated mainly under laboratory or controlled conditions, where calibration, connectivity, and energy supply are stable. Real-world greenhouses, however, face dust accumulation, sensor drift, irregular maintenance, and unstable networks, making results less transferable. This gap underscores the urgent need for multi-site field trials, modular system designs that adapt to diverse environmental conditions, and greater emphasis on practical deployment. Without these efforts, methods that perform well in simulation or controlled tests may not deliver the same reliability in an operational greenhouse [107]. Greenhouse systems often employ heterogeneous sensors, proprietary IoT platforms, and non-uniform data formats, which complicates data fusion and restricts scalability. Without the establishment of open protocols and standardized frameworks, smart greenhouse solutions risk becoming fragmented, vendor-dependent systems with limited long-term applicability [108]. This lack of standardization also hinders meaningful multi-site comparative studies and the development of transferable models across regions.

Although technological efficiency gains are well documented, the economic and social feasibility of advanced systems for small- and medium-scale farmers remains a pressing concern. High costs of sensors, controllers, and cloud subscriptions, combined with limited technical capacity, present adoption barriers in regions most in need of yield improvements. A more critical assessment must recognize the trade-off between high-precision, AI-driven infrastructures and low-cost, low-power alternatives, particularly for resource-constrained farming contexts [109]. Addressing affordability through modular, scalable solutions is as important as pushing the technological frontier. While data filtering and AI-based anomaly detection methods improve reliability, critical issues remain regarding data quality, bias, and security. Many datasets are geographically limited, raising concerns about the generalizability of trained models across climates, crops, and cultural practices. In addition, the increasing reliance on IoT networks exposes greenhouse infrastructures to cybersecurity vulnerabilities, including unauthorized access, data manipulation, and service disruption [110]. Recent advances show that transformer models improve forecasting, lightweight AI makes real-time control possible on edge devices, and blockchain adds security and trust to data management. These tools strengthen IoT frameworks and make smart agriculture more scalable, transparent, and resilient.

The sustainability of smart greenhouse systems must be critically assessed beyond localized gains in water and energy savings. Continuous sensor operation, high-frequency data transmission, and AI-driven computations, particularly under cloud dependency, may elevate overall energy demand, yet life-cycle assessments of hardware, platforms, and data centers remain scarce. Edge computing offers partial relief by decentralizing processing and reducing latency [111], but connectivity gaps, unstable power supply, and scalability constraints of intelligent control algorithms persist as fundamental barriers [112,113]. Equally important are issues of interoperability and trust. Fragmented sensor ecosystems and proprietary IoT platforms limit data fusion and cross-site transferability, while black-box AI models undermine farmer confidence in decision-critical applications such as irrigation and disease detection. Moving toward explainable AI, adaptive learning, and hybrid human–machine frameworks is vital for operational resilience and adoption.

Ultimately, the promise of smart greenhouses lies not only in technological optimization but in modular, affordable, and standardized architectures that balance efficiency with socio-economic feasibility. Hybrid frameworks integrating multi-sensor monitoring, intelligent control, and advanced data processing have shown tangible improvements in resource use and crop productivity [114]. However, scaling these benefits requires an integrated agenda that unites sustainability, interoperability, and farmer-centered design, ensuring that smart greenhouses evolve from experimental deployments into resilient infrastructures for climate-adaptive agriculture.

## 5. Conclusions and Future Directions

This review emphasizes the critical importance of multi-sensor monitoring, intelligent control, and advanced data processing in the sustainable management of smart greenhouses. The integration of diverse environmental sensors enables accurate, real-time observation of temperature, humidity, carbon dioxide, light, and energy usage, while AI-based control techniques such as model predictive control, fuzzy logic, and reinforcement learning enhance environmental stability, crop yields, and resource efficiency. Data filtering methods, ranging from traditional techniques like moving average and Kalman filters to modern AI-driven hybrid models, ensure data reliability by addressing noise, missing values, and variability. Collectively, these technologies provide a foundation for climate-resilient and resource-efficient greenhouse operations.

Despite this progress, a persistent challenge lies in balancing high-precision AI systems with the need for affordable, accessible solutions. Algorithms often require significant computational resources and costly hardware, which can limit adoption by small and medium-scale farms. Addressing this contradiction requires modular system designs that can scale with farm size, lightweight AI models optimized for edge devices, and hybrid frameworks that combine cloud-based precision with localized decision making. These strategies offer a pathway to maintain high performance while reducing costs, ensuring that the benefits of smart greenhouse technologies are broadly attainable.

Looking forward, future research should focus on addressing the technical bottlenecks identified in this review. Priority should be given to developing standardized multi-sensor communication protocols to overcome interoperability barriers, embedding explainable AI in control systems to improve transparency and user trust, conducting comprehensive life cycle assessments to evaluate the long-term sustainability of greenhouse technologies, and implementing blockchain-based frameworks to enhance data ownership, traceability, and security. These research directions are directly linked to challenges of multi-sensor monitoring, intelligent control, and data processing discussed throughout this review.

By aligning innovation with these challenges, smart greenhouse systems can evolve from experimental application to resilient, scalable infrastructures that support climate-adaptive, resource-efficient agriculture. This transition will be crucial in addressing global challenges of food security, resource scarcity, and environmental sustainability in the coming decades.

## Figures and Tables

**Table 1:** Environmental sensors for real-time monitoring in smart greenhouse management.

Category	Sensor Model	Power Consumption (mW)	Data Transfer Range (m)	Accuracy	Applications	Ref.
Temperature sensors	DHT22	~0.3–1.5	~20	±0.5 °C, ±2%	Thermal monitoring	[31,32]
	DS18B20	~7.5	~100	±0.5 °C	Soil heating	[33]
	BME280	~0.012	~1000	±1 °C, ±3%	Environment monitor	[34]
Humidity sensors	SHT31	~5.6	~1000	±2%	Air quality monitoring	[35,36]
	DHT11	~1.5–2	~20	±1°C, ±1%	Basic monitoring setups	[37,38,39]
CO_2_ sensors	MH-Z19B	~300–750	~100	±50 ppm, ±3%	CO_2_ supplementation	[40,41,42]
	Sense Air S8	~150	~200	±70 ppm, ±3%	Plant optimization	[43]
Light sensors	BH1750	~0.36	~1000	±20% (Lux)	LED grow lights	[44,45]
	TSL2591	~1.3	~1000	±10% (Lux)	Solar energy	[25]
Energy sensors	ACS712	~50–60	~500	±1.5%	Energy optimization	[47,48]
	PZEM-004T	~100	~500	±1% (V and I)	Solar power monitor	[49,50]
	INA219	~3–5	~100	±1% (V and I)	Small IoT devices	[51]

**Table 3:** Summary of filtering technologies for smart greenhouses from different studies.

Technology Type	Technology/ Method	Key Parameters/Issues Addressed	Application Scenarios	Impact on Greenhouse Monitoring	Limitations	Ref.
Traditional filters	Moving Average/exponential smoothing/median filters	Noise reduction, signal stability	Basic climate control with moderate sensor noise	Reduces fluctuations, stabilizes temperature/humidity signals	Limited adaptability; poor handling of dynamic/nonlinear changes	[86,87]
	Frequency-domain filters (Low-pass, high-pass, band-pass)	Separation of preferred signals from high-frequency noise	High-interference environments (e.g., fan motors, electrical noise)	Improves signal precision for downstream control	Requires prior knowledge of noise spectrum; limited adaptability	[88]
Statistical/Probabilistic filters	Kalman filter	Dynamic data fusion, anomaly detection, predictive corrections	Multi-sensor fusion for temperature, humidity, and CO_2_	Increases sensor reliability, improves prediction accuracy	Sensitive to model assumptions (linear/Gaussian); requires tuning	[89]
	Particle filter	Handling of nonlinear and non-Gaussian processes	Fault tolerance in distributed sensing; recovery from sensor failures	Enables robust estimation under uncertainty	High computational cost; not ideal for real-time low-power devices	
AI and hybrid methods	AI-powered filtering (ML, DNNs)	Fault detection, anomaly diagnosis, predictive analytics	Large-scale sensor networks with complex patterns	Enables predictive maintenance, decision support	Requires large datasets, high computation	[90,91]
	Kalman + ANN hybrid	Noise reduction, missing data handling, improved prediction accuracy	Environments with strong variability (light, humidity, soil conditions)	Enhances actuator control (e.g., fans, heaters)	Complexity in integration; training required	[92,93,94]
	Ensemble hybrid (ITD + NN)	Improved forecasting, reduced RMSE (Klang: 24%, Langat: 34%)	Energy optimization and climate forecasting	Optimizes resource use, enhances sustainability	Model complexity, parameter sensitivity	[95,96,97]
Edge and cloud-based	Edge computing and cloud analytics	Latency reduction, bandwidth efficiency, anomaly identification	Large-scale smart greenhouse IoT networks	Ensures efficient real-time data handling and resilience	Dependent on connectivity; may raise privacy issues	

## Data Availability

Data is contained within the article.
